# FGF–FGFR Signaling in Parkinson’s Disease: Mechanistic Links to Ferroptosis and Neuroprotection

**DOI:** 10.3390/brainsci16020151

**Published:** 2026-01-29

**Authors:** Hui Wang, Xiao Wen, Min Yan, Ran Li, Dewei Mao, Xuewen Tian

**Affiliations:** 1 Academy of Sports Sciences, Shandong Sport University, Jinan 250102, China; wanghui@stu.sdpei.edu.cn (H.W.); wenxiao@sdpei.edu.cn (X.W.); 202520200585@stu.tjus.edu.cn (M.Y.); liran@sdpei.edu.cn (R.L.); 2 Physical Education Unit, The Chinese University of Hong Kong, Shenzhen 518172, China

**Keywords:** fibroblast growth factors, Parkinson’s disease, ferroptosis, neuroinflammation, neurodegenerative diseases, oxidative stress

## Abstract

Parkinson’s disease (PD) is characterized by progressive degeneration of the nigrostriatal dopaminergic system and α-synuclein (α-syn) pathology, with disease progression driven by convergent mechanisms including neuroinflammation, mitochondrial injury, oxidative stress, and regulated cell-death programs such as ferroptosis. Fibroblast growth factors (FGFs) and fibroblast growth factor receptors (FGFRs) constitute a key signaling system in the central nervous system, influencing not only neuronal survival and glial states but also intersecting with networks governing redox homeostasis and iron metabolism. Accumulating evidence indicates that, beyond classical neurotrophic actions, FGF–FGFR signaling can modulate mitochondrial quality control, glial inflammatory activation, and lipid peroxidation-related processes, thereby reshaping cellular susceptibility to ferroptotic injury. This review summarizes current advances in understanding FGF signaling networks in Parkinson’s disease, synthesizes their potential mechanistic links to the interplay among neuroinflammation, mitochondrial dysfunction, and redox imbalance as well as to ferroptosis regulation, and discusses the experimental basis and translational challenges of targeting the FGF pathway as a disease-modifying therapeutic strategy.

## 1. Introduction

Parkinson’s disease (PD) ranks as the second-most prevalent neurodegenerative disorder of the central nervous system (CNS), surpassed only by Alzheimer’s disease, with over 11 million cases worldwide—a figure projected to increase alongside global demographic aging [1,2,3]. The clinical presentation of PD predominantly involves motor symptoms, including resting tremor, muscular rigidity, bradykinesia, and postural instability. These motor manifestations frequently co-occur with non-motor symptoms, including sleep disturbances, depression, and cognitive decline, which together impair daily functioning. The pathological hallmarks include selective dopaminergic neuron loss in the substantia nigra pars compacta and Lewy body deposition, consisting of misfolded α-synuclein aggregates within surviving neurons [4]. While existing therapies like levodopa provide partial relief for motor symptoms, no interventions currently address the underlying neurodegenerative progression of PD [4].

The pathogenesis of PD involves the interplay of multiple pathological processes, including neuroinflammation with glial activation, α-synuclein aggregation with endoplasmic reticulum (ER) stress, oxidative stress with mitochondrial dysfunction, and ferroptosis [4,5,6,7,8,9]. These processes converge to drive dopaminergic neuronal degeneration and impair neurogenesis. These interconnected mechanisms collectively drive the progressive loss and functional decline of dopaminergic neurons. Chronic neuroinflammation may accelerate disease progression. Microglial overactivation and sustained release of pro-inflammatory cytokines like interleukin-6 (IL-6) and C-X-C motif chemokine ligand 1 (CXCL1) establish a self-perpetuating cycle that worsens neuronal damage and facilitates pathological α-synuclein accumulation [10,11]. Recent studies highlight the critical interaction between ferroptosis and neuroinflammation, now regarded as a fundamental mechanism in PD pathology [12,13]. Therapeutic strategies may therefore need to target both ferroptotic pathways and the neuroimmune microenvironment to reduce neurotoxicity, rebalance iron homeostasis, and slow disease progression [14,15].

The fibroblast growth factor (FGF) family has gained attention as a therapeutic target given its critical involvement in neural development, repair, and homeostasis. With 22 members, this family includes fibroblast growth factor 1 (FGF1, also known as acidic FGF or aFGF), fibroblast growth factor 2 (FGF2, also known as basic FGF or bFGF), and fibroblast growth factor 21 (FGF21), which are abundantly expressed in the adult central nervous system and regulate neuronal survival, autophagy, and inflammatory responses through phosphoinositide 3-kinase/protein kinase B (PI3K/AKT), mitogen-activated protein kinase (MAPK), and extracellular signal-regulated kinase (ERK) pathways [16,17,18]. Emerging evidence suggests that FGFs exhibit neurotrophic properties while indirectly influencing ferroptosis via antioxidant and anti-inflammatory mechanisms. Studies demonstrate that FGF1 and FGF2 enhance the survival of midbrain dopaminergic neurons and reduce α-syn aggregation in PD models induced by 6-hydroxydopamine (6-OHDA), as well as in those induced by 1-methyl-4-phenyl-1,2,3,6-tetrahydropyridine (MPTP) [17,18,19]. FGF21 shows neuroprotective effects in multiple PD models by promoting autophagic flux, mitigating proteasomal dysfunction, and regulating the gut–brain axis [20,21]. Fibroblast growth factor 20 (FGF20) also protects dopaminergic neurons and improves motor function in vitro and in vivo through fibroblast Growth Factor Receptor 1 (FGFR1)activation, highlighting its therapeutic relevance [22].

**Figure 1:**
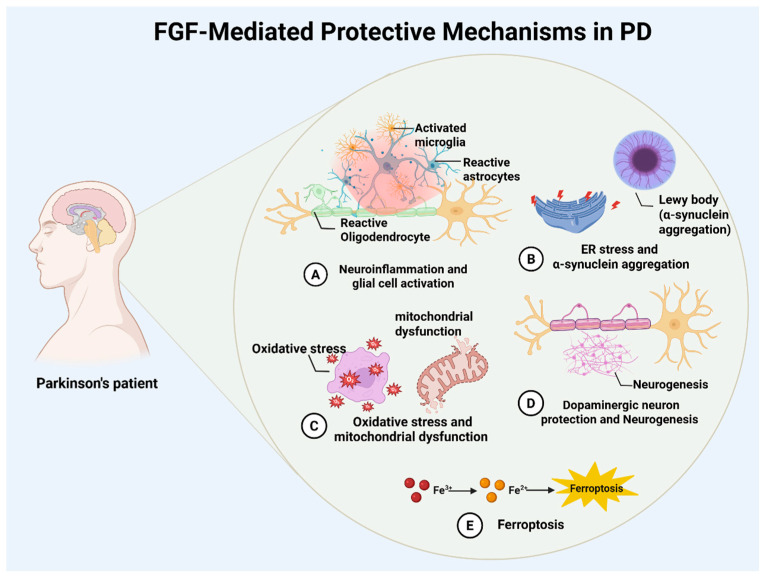
Overview of FGF-mediated neuroprotection in Parkinson’s disease. Schematic summary of reported FGF actions in PD, including modulation of neuroinflammation (A), endoplasmic reticulum stress and α-synuclein pathology (B), antioxidant and mitochondrial pathways (C), dopaminergic neuron survival and neurogenesis (D), and ferroptosis via iron handling and oxidative injury (E).

The FGF family’s multi-target regulatory capacity suggests synergistic neuroprotection in Parkinson’s disease through ferroptosis inhibition and neuroinflammation modulation. Investigating interactions between FGF signaling and ferroptosis pathways could yield novel strategies for neuroprotective and disease-modifying therapies (Figure 1).

## 2. Roles of FGFs in Parkinson’s Disease

### 2.1. Dopaminergic Neuroprotection and Neural Regeneration

FGF family demonstrates robust neuroprotective properties across neurodegenerative disorders, including Alzheimer’s disease (AD) [23,24,25], diabetic encephalopathy [26], and traumatic brain injury (TBI) [27]. FGF21 acts as a metabolic regulator capable of crossing the blood–brain barrier (BBB), where it reinforces BBB integrity and reduces neuroinflammation and oxidative stress by activating the FGFR1–PPARγ pathway and NRF2 antioxidant axis, suggesting therapeutic utility in PD [28,29,30,31]. In AD models, FGF2 suppresses amyloid-β (Aβ) production through β-site APP cleaving enzyme 1 (BACE1) downregulation, augments microglial clearance of Aβ, and restores synaptic plasticity and cognitive function [32,33]. FGF2 also mitigates mutant Htt-mediated cytotoxicity and behavioral impairments in Huntington’s disease (HD) [34], while exhibiting context-dependent regulatory effects in amyotrophic lateral sclerosis (ALS) despite elevated expression levels [35,36,37], highlighting functional divergence among FGF members.

FGF1 and FGF2 show particular promise in PD neuroprotection. FGF1 enhances neuronal viability, neurite extension, and antioxidant defenses via PI3K/AKT and MAPK/ERK pathway activation, regulating Bcl-2 and caspase-3 to support dopaminergic neuron survival and regeneration [17,38,39,40,41,42]. Genetic evidence confirms these neurotrophic effects: NgBR maintains FGF1 expression and PI3K/AKT signaling, with NgBR deficiency impairing neuronal integrity—a phenotype reversible by FGF1 administration, underscoring the NgBR/KAT7/RFX1/FGF1 axis in PD pathogenesis [43]. In MPTP-treated mice, FGF1 restores dopamine metabolism by increasing TH activity and 3,4-dihydroxyphenylacetic acid (DOPAC) levels [44,45] though its efficacy declines with aging [46]. Ex vivo studies demonstrate FGF1’s synergy with dopamine (DA), 12-O-tetradecanoylphorbol-13-acetate (TPA), and 3-isobutyl-1-methylxanthine (IBMX) in inducing TH expression and dopaminergic differentiation [47,48], outperforming FGF2 in generating TH-positive NB69 cells with elevated dopamine/norepinephrine levels [49]. The C-terminal domain of FGF1 further confers anti-apoptotic effects by inhibiting p53-mediated mitochondrial pathways [50].

FGF2 maintains neuroprotective activity despite reduced substantia nigra expression in PD patients, with preserved FGFR-1 localization in surviving dopaminergic neurons implying pathological FGF signaling activation [51]. Colocalization studies in rodents, primates, and humans reveal parallel declines in FGF2 and TH-positive neurons following 6-OHDA lesions [52]. Genetic ablation exacerbates 6-OHDA sensitivity without compensatory FGF upregulation, whereas transgenic FGF2 overexpression enhances protection [53,54]. Mechanistically, FGF2 sustains neuronal integrity by suppressing caspase-3 through PI3K/AKT/Bcl-2 signaling, conferring resistance to MPP^+^ toxicity [42]. Collaborative effects with brain-derived neurotrophic factors (BDNFs) enable FGF2 to drive dopaminergic differentiation of mesenchymal stem cells [55,56], though optimal neuroprotection often requires combination with other trophic factors [57]. FGF20 similarly promotes dopaminergic survival via FGFR1/MAPK activation and, when combined with Wnt family member 5A (WNT5A) and FGF2, improves the differentiation and integration of stem cell-derived dopaminergic precursors [22,58].

**Figure 2:**
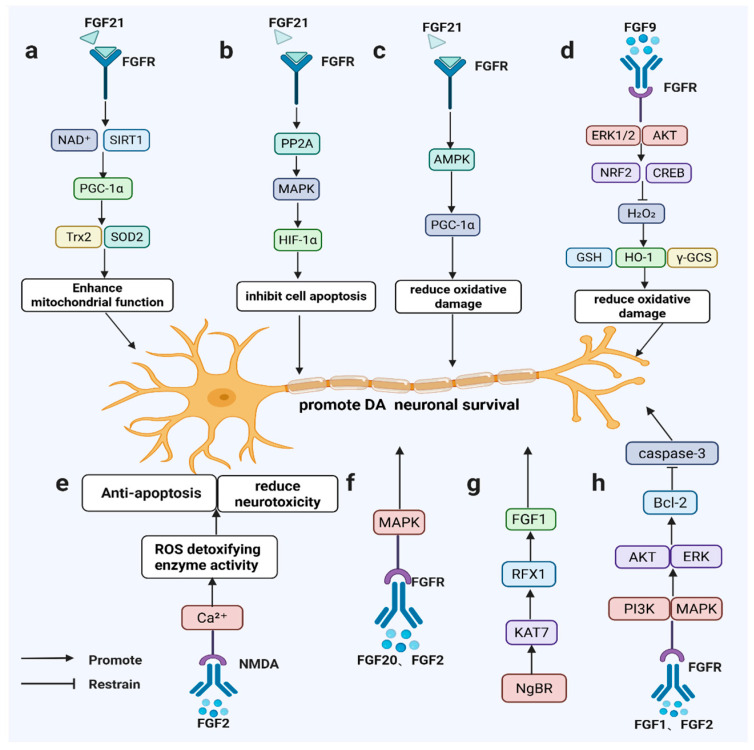
FGF signaling promotes dopaminergic neuron survival in PD-relevant models. FGF21 activates SIRT1/HIF-1α/AMPK–PGC-1α (a–c); FGF9 induces ERK/AKT–NRF2/CREB antioxidant responses (d); FGF2 modulates NMDA/Ca^2^⁺ signaling and ROS detoxification (e); FGF20/FGF2 signal via FGFR–MAPK (f); NgBR–KAT7–RFX1 maintains FGF1 expression (g); and FGF1/FGF2 activate PI3K/AKT and ERK to upregulate Bcl-2 and inhibit caspase-3 (h).

Beyond neuroprotection, FGF members regulate neurogenic processes. FGF2 preserves dopaminergic neurons by modulating N-methyl-D-aspartate (NMDA) receptors, calcium homeostasis, and ROS detoxification [59,60], while stimulating hippocampal and midbrain neurogenesis to repair damaged dopaminergic circuits [60,61]. FGF20 efficiently generates TH-positive neurons from stem cells but requires cooperative signaling for sustained functional recovery in adult brains [62] (Figure 2).

### 2.2. FGFs, Iron Homeostasis, and Ferroptosis in PD

Dysregulated iron homeostasis and ferroptosis are considered major pathological bases for dopaminergic (DA) neuron degeneration in the substantia nigra in PD []. Iron overload exacerbates cellular injury by accelerating lipid peroxidation and generating large amounts of reactive oxygen species (ROS) and malondialdehyde (MDA) [63]. In particular, Nuclear Receptor Coactivator 4 (NCOA4)-mediated ferritinophagy delivers Ferritin Heavy Chain 1 (FTH1) to autophagosomes and promotes its lysosomal degradation, thereby releasing labile iron; when aberrantly overactivated, this process can drive intracellular iron accumulation and amplify lipid peroxidation and ferroptosis-related signaling [64].

Accumulating evidence suggests that certain members of the FGF family may attenuate oxidative damage and cellular vulnerability under iron-overload conditions by strengthening antioxidant defenses and/or modulating processes related to iron homeostasis. Notably, FGF21 has been implicated as a regulator of ferroptosis. In iron-overload settings, FGF21 has been reported to activate nuclear factor erythroid 2–related factor 2 (NRF2) signaling and modulate Heme Oxygenase-1 (HO-1) turnover, thereby alleviating cellular injury [65]. Reduced NRF2 and FGF21 levels may therefore weaken iron-homeostatic control and anti-ferroptotic defenses [65].In addition, FGF21 can bind to the heavy and light chains of ferritin to prevent excessive degradation, thereby maintaining intracellular iron balance and mitigating tissue damage caused by iron overload [66]. Moreover, FGF21 promotes autophagy and antioxidant responses via sirtuin 1 (SIRT1) activation and reduces α-syn aggregation, thereby indirectly lowering the risk of neuronal injury [67]. Acidic fibroblast growth factor (aFGF), also known as fibroblast growth factor 1 (FGF1), plays an important role in homeostatic regulation in PD. Zhong et al. [68] reported that FGF1 suppresses ER stress-induced upregulation of tribbles homolog 3 (TRB3), modulates the mechanistic target of rapamycin (mTOR)/p62 pathway, and activates autophagy, thereby improving DA neuronal injury and motor dysfunction. Furthermore, JWA (also known as ARL6IP5 (ADP-ribosylation factor-like 6 interacting protein 5)) can bind NCOA4 to inhibit ferritinophagy, reduce labile iron release and lipid peroxidation, and alleviate neuronal degeneration in PD models [69]. Similarly, ganoderic acid A was shown to attenuate ferroptosis in nigral DA neurons in mice by inhibiting NCOA4-mediated ferritinophagy [64].Our integrated single-nucleus RNA sequencing and spatial transcriptomics analyses in a PD mouse model indicate that astrocyte/oligodendrocyte-associated FGF signaling may contribute to ferroptosis regulation. In the PD glial microenvironment, FGF signaling is suppressed and is accompanied by upregulated Ca^2+^-related signatures and attenuation of the NRF2/SLC7A11/GPX4 antioxidant axis, consistent with impaired glial redox support and a consequent increase in neuronal susceptibility to ferroptosis [70]. In neurons, FGF2 has also been implicated in Ca^2+^ regulation; 24 h bFGF/FGF2 treatment has been reported to modestly increase intracellular Ca^2+^ in cultured primary hippocampal neurons, as assessed by Fura-2 calcium imaging [71].

Specifically, FGF signaling is suppressed in the PD microenvironment, accompanied by a marked elevation of Ca^2+^ and attenuation of the NRF2/solute carrier family 7 member 11 (SLC7A11; xCT)/ glutathione peroxidase 4 (GPX4) antioxidant axis, thereby increasing neuronal susceptibility to ferroptosis []. In addition, upregulation of FGF1 in OL may suppress ferroptosis by modulating Ast–OL communication and influencing inflammatory responses and myelin homeostasis []. These findings provide a testable mechanistic hypothesis and research framework to elucidate how weakened FGF signaling, through glial interactions, shapes lipid peroxidation and ferroptosis vulnerability and ultimately contributes to dopaminergic neuronal injury. Early evidence has shown that FGF1 protects neuroblastoma (SH-SY5Y) cells from p53 (a key gene in ferroptosis)-dependent cell death [50]. More recent studies further clarified upstream regulatory mechanisms of FGF1 transcription: Nogo-B receptor (NgBR) deficiency suppresses FGF1 transcription via the lysine acetyltransferase 7 (KAT7)/ regulatory factor X1 (RFX1) pathway, leading to PI3K/AKT inactivation and neuronal injury, whereas exogenous FGF1 treatment partially restores AKT activity and mitigates this injury [43], suggesting that FGF1 may have substantial protective potential against iron-related neuronal damage. In parallel, FGF9 may also indirectly modulate PD-related ferroptosis by enhancing antioxidant defenses: FGF9 upregulates HO-1 and γ-glutamylcysteine ligase (GCL), activates ERK1/2 and AKT pathways, and, through cAMP response element–binding protein (CREB) and NRF2-mediated transcription of antioxidant genes, attenuates MPTP-induced oxidative stress and structural damage in DA neurons [72].

**Figure 3:**
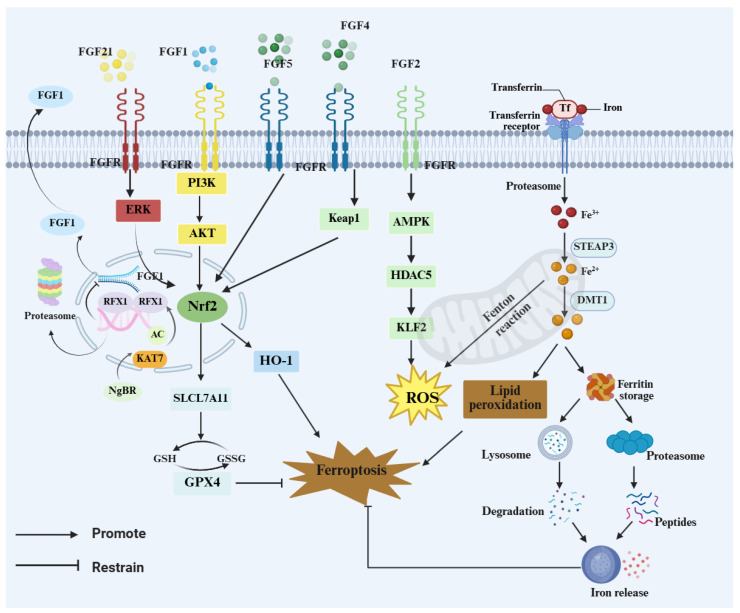
FGF–FGFR signaling restrains ferroptosis via NRF2 programs and iron handling. FGFs (FGF1/2/4/5/21) activate ERK, PI3K–AKT, and AMPK signaling to engage the Kelch-like ECH-associated protein 1 (KEAP1)–NRF2 axis, downstream targets such as inducing HO-1 and SLC7A11 and maintaining the GSH–GPX4 defense to limit ROS and lipid peroxidation. Transferrin/transferrin receptor (Tf/TfR)-mediated iron uptake expands the labile Fe^2+^ pool and drives Fenton chemistry, whereas ferritin turnover via lysosomes/proteasomes releases iron and reinforces ferroptotic cascades.

This section highlights dysregulated iron homeostasis and ferroptosis as key drivers of nigral dopaminergic neurodegeneration in Parkinson’s disease. Enhanced NCOA4-mediated ferritinophagy can expand the labile iron pool, thereby promoting lipid peroxidation and ferroptosis-associated injury. Evidence suggests that FGF1, FGF9, and FGF21 may support iron homeostasis and strengthen NRF2-linked antioxidant defenses, including the GSH–GPX4 axis (Figure 3). Omics data further link impaired FGF signaling to attenuation of the NRF2/SLC7A11/GPX4 program, suggesting that FGF–FGFR signaling may influence ferroptosis susceptibility through both neuron-intrinsic defenses and glia-mediated microenvironmental regulation. In neurons, FGF signaling may limit lipid peroxidation through NRF2-dependent transcription and preservation of the GSH–GPX4 defense. In glia, FGF–FGFR activity may indirectly shape neuronal oxidative and iron burden by influencing inflammatory tone, redox balance, and iron metabolism-related processes. These effects may vary by cell type, disease stage, and model. Cell-type-specific and temporally controlled causal studies are needed to define the key FGF–FGFR axes and their stage-dependent actions.

### 2.3. The Role of Fibroblast Growth Factor in Neuroinflammation in PD

#### 2.3.1. Anti-Inflammatory Effects of FGF in Neurodegenerative Diseases

Neuroinflammation in PD accelerates neuronal degeneration through proinflammatory cytokine release [73,74]. Fibroblast growth factor 21 (FGF21) exhibits anti-inflammatory and neuroprotective effects in neurological disorders such as Alzheimer’s disease, where it mitigates inflammation and oxidative stress by activating the FGFR1c (the IIIc isoform of FGFR1) and AMPK/MAPK pathways [75]. Studies in diabetic and aging models further show that FGF21 improves mitochondrial function by inhibiting nuclear factor kappa B (NF-κB) activity and reducing proinflammatory cytokine production [18,76]. In diabetic stroke and high-fat diet models, FGF21 alleviates metabolic dysfunction and neuroinflammation, facilitating cognitive recovery and neural repair [77,78,79]. Like FGF21, fibroblast growth factor 2 (FGF2) promotes neurorepair by reducing inflammation and enhancing neuronal regeneration when administered during early stages [80,81].

#### 2.3.2. Roles of FGFs in Glial Activation and Neuroprotection in PD

In PD, complex inflammatory crosstalk occurs between microglia, astrocytes, oligodendrocytes, and dopaminergic neurons. Elevated inflammatory responses appear in both central and peripheral systems of PD patients, particularly in brain tissue, blood, and gut, where they correlate with α-synuclein pathology and neurodegeneration [76,82]. Microglial activation spans a spectrum that is often simplified into M1-like (pro-inflammatory) and M2-like (anti-inflammatory) phenotypes. M1 microglia release tumor necrosis factor-α (TNF-α), interleukin-1β (IL-1β), and IL-6 to drive neuroinflammation, while M2 microglia produce IL-4 and IL-10 to mediate immunosuppression [83]. During PD progression, hyperactivated microglia secrete C-X-C motif chemokine ligand 12 (CXCL12), interferon-gamma (IFN-γ), and IL-6, amplifying neuronal injury [84].

Beyond downstream cell-intrinsic cascades, paracrine FGF crosstalk is also shaped by membrane-proximal and cell-surface events that control extracellular ligand availability. These membrane-proximal steps determine when and where extracellular FGF2 becomes available to modulate glial activation. FGF2 lacks a signal peptide and is exported via an unconventional secretion route initiated by PI(4,5)P_2_-dependent oligomerization at the inner plasma membrane leaflet. Recent evidence further indicates that transbilayer asymmetry of PI(4,5)P_2_ accelerates pore formation and FGF2 membrane translocation, while at the outer leaflet, FGF2 oligomers are captured and disassembled by the heparan sulfate proteoglycan glypican-1 (GPC1), enabling cell-surface presentation and engagement in ternary signaling complexes [85,86,87]. FGF2 inhibits microglial activation through CD200 signaling, downregulating IL-1β, intercellular adhesion molecule 1 (ICAM-1), and cluster of differentiation 86 (CD86) expression. This growth factor also stimulates neuronal CD200 production via ERK pathway activation, reinforcing CD200/CD200R-mediated anti-inflammatory signaling [88,89,90].

Reactive astrocytes and microglia contribute to PD pathogenesis by releasing glutamate and inflammatory mediators, which exacerbate excitotoxicity and neuroinflammation [91,92]. Astrocytes in 6-OHDA-induced PD models markedly increase FGF2 and FGFR2 expression, facilitating stress adaptation and neuroprotective repair [93,94]. FGF20 interacts with FGFR1, FGFR3, and FGFR4 to activate RAS/MAPK and PI3K/AKT pathways, enhancing dopamine production while counteracting 6-OHDA and oxidative damage [95,96]. Oligodendrocytes in PD upregulate FGF1, potentially mediating astrocyte–oligodendrocyte communication to modulate inflammation, myelin maintenance, and ferroptosis resistance [70].

FGF2 impairs oligodendrocyte maturation by suppressing myelin-related genes and modifying Fibroblast Growth Factor Receptor (FGFR) expression patterns. FGFR signaling disrupts myelin protein trafficking, and FGF2 depletion enhances oligodendrocyte regeneration, confirming its inhibitory effect on remyelination [97,98,99]. Fibroblast growth factor 10 (FGF10) attenuates microglial and macrophage activation by inhibiting Toll-like receptor 4 (TLR4)/ myeloid differentiation primary response 88 (MyD88)/NF-κB signaling, which reduces TNF-α and IL-6 production, while PI3K/AKT activation promotes glial cell viability [100,101]. Through astrocyte-mediated mechanisms, FGF2 sustains dopaminergic neuron survival and coordinates inflammatory resolution via transforming growth factor-β (TGF-β) secretion [102,103,104,105,106].

#### 2.3.3. Neuroinflammation Modulation and Neuroprotective Effects of FGFs in PD Models

**Figure 4:**
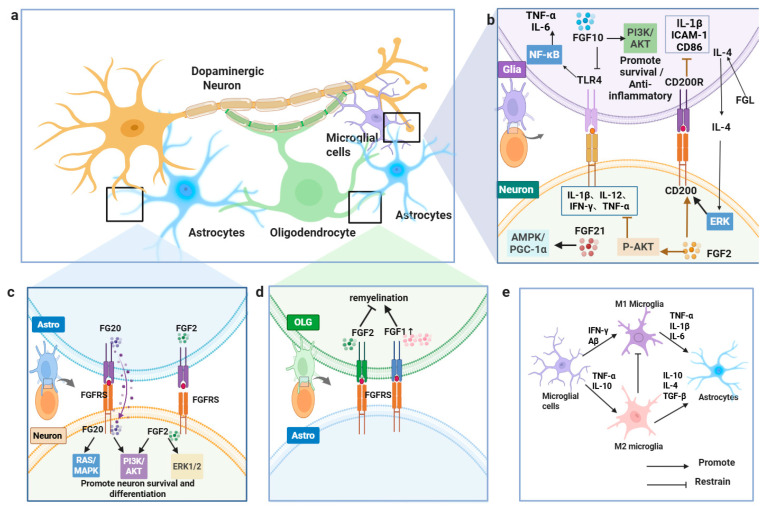
**FGFs regulate glia–neuron interactions in PD.** (**a**) Overview of glia–neuron crosstalk. (**b**) FGF2/FGF10/FGF21 engage PI3K–AKT, ERK, and AMPK–PGC-1α to limit inflammation and support survival. (**c**) FGF20/FGF2 activate FGFR signaling to promote neuronal survival/differentiation. (**d**) FGFs facilitate oligodendrocyte remyelination. (**e**) FGFs modulate microglial states and astrocyte signaling.

In 1-methyl-4-phenylpyridinium (MPP^+^)-induced SH-SY5Y cells, primary dopaminergic neuron models, and MPTP-induced PD mouse models, FGF21 and FGF2 exhibit neuroprotective, antioxidant, and anti-inflammatory properties through FGFR binding and downstream signaling pathway activation [107,108]. FGF21 enhances mitochondrial function via the AMPK/PGC-1α pathway, decreases ROS generation, mitigates NADPH oxidase (NOX)- and inducible nitric oxide synthase (iNOS)-mediated oxidative stress, and reduces neurotoxicity. It also attenuates inhibitor of κB alpha (IκBα) phosphorylation, prevents NF-κB nuclear translocation, and markedly lowers IL-1β, IL-12, IFN-γ, and TNF-α expression, suppressing microglial and astrocytic activation [107]. FGF2 supports neuronal survival and differentiation by stimulating the ERK1/2 and PI3K/AKT pathways, inhibits Bcl-2–associated agonist of cell death (Bad), and bolsters neuronal viability through mTOR activation [109,110]. In MPTP-induced PD models, FGF2 elevates AKT phosphorylation, curbs necroptosis and inflammatory signaling, reduces astrocyte activation, and preserves dopaminergic neurons. Furthermore, it regulates inflammatory cytokines, including IL-10, CX3CL1, IL-1β, IL-6, and TNF-α, restoring microglial-mediated neuronal immune surveillance [111,112,113]. While FGFs show promise in preclinical PD models, their therapeutic potential warrants further clinical exploration (Figure 4).

Current evidence supports two interrelated modes of FGF–FGFR action in Parkinson’s disease. First, neuron-intrinsic FGF–FGFR signaling can promote cell survival and proteostasis. Second, glia-mediated FGF–FGFR signaling can shape the local microenvironment by modulating inflammatory programs, redox homeostasis, and iron-handling processes. These components are not mutually exclusive; rather, they are mechanistically coupled through bidirectional glia–neuron communication. However, cell-type-resolved causal evidence remains limited, making it difficult to determine whether stage-specific effects are predominantly driven by neuron-intrinsic versus glia-mediated FGF–FGFR signaling. To more precisely disentangle their relative contributions to ferroptosis-associated pathology, future studies could adopt single-cell causal frameworks such as in vivo Perturb-seq [114], which enables AAV-delivered, programmable genetic perturbations with transcriptomic readouts at single-cell resolution in intact brain tissue, thereby providing cell-type-specific causal evidence for prioritized FGF–FGFR axes and their stage-dependent effects.

### 2.4. ER Stress and α-Synuclein Aggregation in PD

#### 2.4.1. The FGF Family Attenuates ER Stress and Reduces α-Synuclein Toxicity

Proteostasis disruption plays a central role in neuronal damage during PD and other neurodegenerative disorders. Neurons exhibit heightened susceptibility to misfolded or mutant proteins owing to their elevated metabolic demands and restricted regenerative potential. In PD, pathological α-synuclein (α-syn) oligomers accumulate in the ER, inducing severe endoplasmic reticulum stress [115]. The unfolded protein response (UPR) mobilizes protein kinase R-like ER kinase (PERK), inositol-requiring enzyme 1 (IRE1), and activating transcription factor 6 (ATF6) signaling pathways to reestablish proteostasis by modulating protein folding, translational suppression, and misfolded protein clearance. When ER stress persists or UPR mechanisms fail, binding immunoglobulin protein (BiP; also known as GRP78) dissociation initiates pro-apoptotic cascades through PERK- eukaryotic initiation factor 2α (eIF2α) activation, driving caspase-mediated neuronal death and accelerating PD progression [115]. Phosphorylation at Ser129 augments α-syn’s neurotoxic and aggregation-prone properties. Rotenone-mediated mitochondrial impairment elevates Ser129 phosphorylation, facilitating α-syn ER accumulation, UPR induction, and subsequent neuronal apoptosis [116].Postmortem analyses confirm colocalization of phosphorylated α-syn aggregates with UPR markers in PD brains, underscoring their pathogenic significance [117,118,119].

During early PD stages, ER-localized α-syn aggregates correlate with disease severity. Salubrinal diminishes ER α-syn accumulation, further implicating ER dysfunction in PD pathogenesis [117].The ubiquitin–proteasome system eliminates soluble α-syn, whereas autophagy–lysosomal pathways degrade insoluble aggregates. Under proteotoxic stress, autophagy activation occurs via transcription factor EB (TFEB) nuclear translocation, which enhances autophagic flux and mitigates dopaminergic neuron toxicity [120,121].

#### 2.4.2. FGF Enhances Mitochondrial Function and Clears α-Synuclein

The FGF family has emerged as a key regulator in mitigating ER stress, augmenting autophagy, and counteracting α-syn toxicity. Acidic FGF (FGF1, aFGF) and basic FGF (FGF2, bFGF) have demonstrated robust neuroprotective properties across various Parkinson’s disease (PD) models. aFGF suppresses α-syn aggregation and preserves tyrosine hydroxylase (TH)-positive neuronal viability by activating PI3K/AKT and ERK1/2 signaling, which concurrently downregulates ER stress markers GRP78, C/EBP homologous protein (CHOP), and Caspase-12, leading to improved motor function []. bFGF similarly enhances PI3K/AKT signaling to inhibit apoptosis, an effect attenuated by pathway-specific inhibitors, confirming mechanistic specificity [122]. Notably, proteome-wide profiling in bFGF-treated hippocampal neurons further suggests a context-dependent facet of bFGF/FGF2 signaling, including increased extracellular vesicle release and remodeling of Lewy body pathology-associated protein networks that contain α-syn-interacting modules, indicating that the net impact of bFGF may vary with disease stage and pathological burden [123]. Bcl-2-associated X protein (Bax) and B-cell lymphoma 2 (Bcl-2) are key regulators of apoptosis. This pathway further modulates apoptosis by promoting Bax phosphorylation and increasing Bcl-2 expression [124].

**Figure 5:**
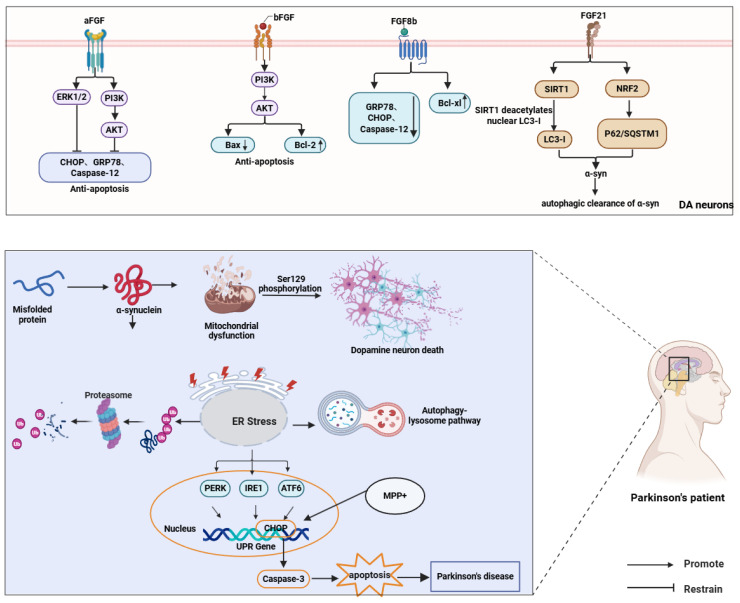
FGFs protect dopaminergic neurons in PD by attenuating ER stress and apoptosis and promoting autophagic clearance of α-syn. FGF1/FGF2/FGF8b activate PI3K–AKT/ERK signaling, whereas FGF21 enhances autophagy via SIRT1-mediated deacetylation of nuclear LC3-I and NRF2-associated signaling.) ↑ and ↓ denote increased and decreased protein expression, respectivel.

FGF8b reduces ER stress by suppressing Caspase-12, GRP78, and Bax while upregulating the anti-apoptotic factor Bcl-xl [125]. FGF21, an endocrine regulator, has recently emerged as a neuroprotective agent in PD. It sustains dopaminergic neuron homeostasis through mitochondrial stabilization, ER stress mitigation, and neuroinflammatory suppression, with its expression governed by ATF4 during autophagy-impaired ER stress [126,127]. FGF21 attenuates α-syn accumulation and neuronal degeneration via the SIRT1-autophagy axis, where SIRT1-mediated microtubule-associated protein 1 light chain 3 (LC3) deacetylation enhances autophagic clearance [67]. Furthermore, FGF21 elevates LC3-II and Beclin-1 levels to accelerate toxic protein removal while activating autophagy-related genes NRF2 and p62/SQSTM1, forming a self-reinforcing cycle [20,128] (Figure 5).

### 2.5. Oxidative Stress and Mitochondrial Dysfunction

Oxidative stress and mitochondrial dysfunction are central to PD pathogenesis. Nutritional deficiencies in PD patients often impair antioxidant defenses, accelerating neuronal degeneration. The endocrine factor FGF21 has emerged as a key neuroprotective agent, with studies showing its ability to improve mitochondrial function via AMPK/PGC-1α signaling, attenuate oxidative stress, suppress neuroinflammation, and promote neuronal survival and repair [107]. FGF21 also counteracts MPTP-induced declines in mitochondrial DNA copy number and restores electron transport chain gene expression, reinforcing its role in mitochondrial homeostasis [107]. Chen et al. demonstrated that FGF21 reduces neurotoxicity, prevents apoptosis, decreases tau hyperphosphorylation, and alleviates oxidative stress in vitro and in vivo by regulating the protein phosphatase 2A (PP2A)/MAPK/ hypoxia-inducible factor 1α (HIF-1α) axis [129]. In dopaminergic neurons, FGF21 increases nicotinamide adenine dinucleotide (NAD^+^) and SIRT1 levels, activates PGC-1α, and upregulates antioxidant enzymes such as thioredoxin 2 (Trx2) and superoxide dismutase 2 (SOD2), enhancing mitochondrial respiration and cell survival [130]. Its expression in glial cultures further suggests paracrine neuroprotective effects [130,131].

FGF21 serves as a serum biomarker for mitochondrial disorders, often paired with growth differentiation factor 15 (GDF-15) to evaluate dysfunction severity [132]. In mitochondrial myopathies, muscle FGF21 correlates with cytochrome c oxidase (COX)-negative fiber counts, while elevated serum levels mirror phenotypes seen in transgenic FGF21-overexpressing mice [133]. In obesity and insulin resistance models, FGF21 improves peripheral insulin sensitivity, restores brain mitochondrial function, enhances synaptic plasticity, and prevents neuronal apoptosis and cognitive impairment [134]. It also activates autophagy in PD proteasome inhibition models, reducing cytotoxicity from protein aggregates [20]. FGF21 modulates gut microbiota and metabolic homeostasis, suppresses A-type K^+^ currents in substantia nigra neurons, and improves hippocampal long-term potentiation (LTP) and motor coordination in mice. High-dose FGF20 lowers serum malondialdehyde and boosts antioxidant activity [21]. Similarly, FGF9 reduces H_2_O_2_ levels in cortical neurons, elevates GSH, and upregulates HO-1 and γ-glutamylcysteine synthetase (γ-GCS), mitigating oxidative damage [135]. This neuroprotection involves coordinated ERK1/2 and AKT activation upon FGFR stimulation, which enhances NRF2 and CREB transcriptional activity to upregulate antioxidant genes and shield dopaminergic neurons from MPP^+^ toxicity [72].

## 3. Conclusions and Prospects

In summary, FGF–FGFR signaling shows regulatory potential across major PD processes, including neuroinflammation, cellular stress responses, iron dyshomeostasis and ferroptosis, as well as neuroprotection and regeneration. Available evidence is more consistent with a cross-cell-type, network-based mode of action—operating through multiple coordinated pathways—than with protection restricted to a single cell-intrinsic mechanism. Importantly, the impact of FGF–FGFR signaling should be viewed as pathology state-dependent rather than uniformly protective, varying with disease stage, pathological burden, and microenvironmental context. From a translational perspective, intranasal delivery of FGF21-overexpressing mesenchymal 72stem cells confers neuroprotection in PD animal models and is associated with activation of the pro-survival AKT–BDNF–Bcl-2 axis, improved mitochondrial function, and attenuation of dopaminergic neurodegeneration [136]. In parallel, advances in the structural characterization of FGFR signaling complexes and ligand selectivity provide a methodological foundation for engineering paracrine FGFs, enabling biased signaling and selective tuning of downstream outputs while reducing potential mitogenic risk [137].

To prioritize candidate axes, we propose four criteria: (i) achievable CNS exposure, (ii) safety (mitogenic/off-target proliferative signaling and, under high pathological burden, potential network-level liabilities such as EV-facilitated propagation of synucleinopathy-related factors), (iii) alignment with stage-/phenotype-specific PD biology, and (iv) pharmacodynamic readouts for target engagement and pathway modulation. The endocrine FGF21–FGFR1c/β-Klotho axis appears particularly promising, whereas paracrine FGFs may require brain-targeted delivery and receptor-selective/biased designs to improve selectivity. Proteomics in bFGF/FGF2-treated hippocampal neurons reported increased EV release and remodeling of Lewy body pathology-associated protein networks (including α-syn modules), underscoring that paracrine FGF outputs may shift with burden and stage and warrant validation in dopaminergic circuits and human-relevant systems. Going forward, biomarker-informed, stage-stratified PK/PD frameworks with cell-type- and time-resolved validation are needed to define actionable FGFR subtypes, therapeutic windows, and downstream nodes that balance efficacy with safety.

Nevertheless, substantial barriers remain in translating protective effects observed in experimental models into reproducible clinical benefit. First, dominant pathological drivers across PD stages and subtypes have not been fully resolved, limiting rigorous definition of therapeutic windows, target populations, and cellular targets. Second, despite improved targeting and bioavailability achieved by emerging delivery platforms, blood–brain barrier penetration remains a key limitation; further optimization is required to achieve reliable CNS delivery. Third, to improve selectivity and safety of FGF-targeted interventions, structure-guided design exploiting differences among FGFR subtypes is needed, and targeted protein degradation strategies such as PROTACs warrant evaluation to eliminate aberrant receptors or signaling components. Fourth, interspecies differences in FGFR/Klotho expression profiles and microenvironmental context may constrain extrapolation, necessitating validation of key receptor axes in human-relevant systems and clinically relevant models. Future studies should integrate cell-type-specific models with brain-targeted delivery technologies, develop FGF analogs or FGFR modulators, and establish quantitative endpoints to rigorously evaluate disease-modifying potential. Given stage dependence, quantitative pathology indices and biomarker-based stratification are essential to define windows and monitor efficacy and safety.

## Data Availability

No new data were created or analyzed in this study. Data sharing is not applicable to this article.

## Abbreviations

6-OHDA, 6-hydroxydopamine; Aβ, amyloid-β; AD, Alzheimer’s disease; AKT, protein kinase B; ALS, amyotrophic lateral sclerosis; AMPK, AMP-activated protein kinase; ARL6IP5, ADP-ribosylation factor-like 6 interacting protein 5; Ast, astrocytes; ATF4, activating transcription factor 4; ATF6, activating transcription factor 6; Bad, Bcl-2–associated agonist of cell death; BACE1, β-site APP cleaving enzyme 1; Bax, Bcl-2–associated X protein; BBB, blood–brain barrier; Bcl-2, B-cell lymphoma 2; Bcl-xL, B-cell lymphoma-extra large; BDNF, brain-derived neurotrophic factor; BiP, binding immunoglobulin protein (also GRP78); Ca^2+^, calcium ion; CD86, cluster of differentiation 86; CD200R, CD200 receptor; CHOP, C/EBP homologous protein; CNS, central nervous system; COX, cytochrome c oxidase; CREB, cAMP response element–binding protein; CX3CL1, C-X3-C motif chemokine ligand 1; CXCL1, C-X-C motif chemokine ligand 1; CXCL12, C-X-C motif chemokine ligand 12; DA, dopamine; DA neuron, dopaminergic neuron; DOPAC, 3,4-dihydroxyphenylacetic acid; eIF2α, eukaryotic initiation factor 2α; ER, endoplasmic reticulum; ERK, extracellular signal-regulated kinase; FGF, fibroblast growth factor; FGFR, fibroblast growth factor receptor; FGFR1c, FGFR1 IIIc isoform; FTH1, ferritin heavy chain 1; GCL, γ-glutamylcysteine ligase; GDF-15, growth differentiation factor 15; GPX4, glutathione peroxidase 4; GRP78, glucose-regulated protein 78 (alias of BiP); GSH, glutathione; HD, Huntington’s disease; HIF-1α, hypoxia-inducible factor 1α; HO-1, heme oxygenase-1; Htt, huntingtin; IBMX, 3-isobutyl-1-methylxanthine; ICAM-1, intercellular adhesion molecule 1; IFN-γ, interferon-gamma; IκBα, inhibitor of κB alpha; IL-1β, interleukin-1β; IL-4, interleukin-4; IL-6, interleukin-6; IL-10, interleukin-10; IL-12, interleukin-12; iNOS, inducible nitric oxide synthase; IRE1, inositol-requiring enzyme 1; JWA, JWA protein (alias ARL6IP5); KAT7, lysine acetyltransferase 7; Keap1, Kelch-like ECH-associated protein 1; LC3, microtubule-associated protein 1 light chain 3; LTP, long-term potentiation; MAPK, mitogen-activated protein kinase; MDA, malondialdehyde; MPP^+^, 1-methyl-4-phenylpyridinium; MPTP, 1-methyl-4-phenyl-1,2,3,6-tetrahydropyridine; mTOR, mechanistic target of rapamycin; MyD88, myeloid differentiation primary response 88; NAD^+^, nicotinamide adenine dinucleotide; NCOA4, nuclear receptor coactivator 4; NF-κB, nuclear factor kappa B; NgBR, Nogo-B receptor; NMDA, N-methyl-D-aspartate; NOX, NADPH oxidase; NRF2, nuclear factor erythroid 2–related factor 2 (NRF2); OL, oligodendrocytes; p62/SQSTM1, sequestosome 1; PD, Parkinson’s disease; PERK, protein kinase R-like ER kinase; PGC-1α, peroxisome proliferator-activated receptor gamma coactivator 1-alpha; PI3K, phosphoinositide 3-kinase; PK/PD, pharmacokinetics/pharmacodynamics; PP2A, protein phosphatase 2A; PPARγ, peroxisome proliferator-activated receptor gamma; PROTAC, proteolysis targeting chimera; RAS, rat sarcoma (Ras) small GTPase; RFX1, regulatory factor X1; ROS, reactive oxygen species; Ser129, serine 129; SH-SY5Y, human neuroblastoma cell line; SIRT1, sirtuin 1; SLC7A11, solute carrier family 7 member 11; SOD2, superoxide dismutase 2; Tf, transferrin; TfR, transferrin receptor; TGF-β, transforming growth factor beta; TH, tyrosine hydroxylase; TLR4, Toll-like receptor 4; TNF-α, tumor necrosis factor alpha; TPA, 12-O-tetradecanoylphorbol-13-acetate; TRB3, tribbles homolog 3; Trx2, thioredoxin 2; UPR, unfolded protein response; WNT5A, Wnt family member 5A; xCT, cystine/glutamate antiporter light chain (system x_c_^−^ ; encoded by SLC7A11); α-syn, α-synuclein.
